# Increased Oxidative Stress Induced by *Rubus* Bioactive Compounds Induce Apoptotic Cell Death in Human Breast Cancer Cells

**DOI:** 10.1155/2019/6797921

**Published:** 2019-06-03

**Authors:** Blassan P. George, Heidi Abrahamse

**Affiliations:** Laser Research Centre, Faculty of Health Sciences, University of Johannesburg, Doornfontein, 2028 Johannesburg, South Africa

## Abstract

Bioactive compounds from plants represent good candidate drugs for the prevention and treatment of various forms of cancer. Berries are rich sources of bioactive compounds, and there has been an increasing interest in the study of therapeutic action of wild berries. Oxidants are generated continuously in biological system as a result of physiological process. When there is an imbalance between oxidants and antioxidants, it leads to a condition called oxidative stress. Natural compounds as inducers of oxidative stress are able to modulate the physiological functions of cancer cells leading to cell death or survival. The aim of this study was to evaluate the induction of apoptosis by isolated bioactive compounds (1-(2-hydroxyphenyl)-4-methylpentan-1-one (C1) and 2-[(3-methylbutoxy) carbonyl] benzoic acid (C2)) from *Rubus fairholmianus* against MCF-7 breast cancer cells. The exposure of C1 and C2 reduced viability (IC_50_ of C1: 4.69; C2: 8.36 *μ*g/mL) and proliferation. Cytochrome c release from mitochondria and changes in mitochondrial membrane potential of treated cells supported the intrinsic apoptotic cell death. Reactive oxygen species (ROS) production after treatment with C1 and C2 was found to be higher and induced nuclear damage. Expression of apoptotic proteins after the treatments was significantly upregulated as indicated using immunofluorescence (caspase 9, p53, and Bax), western blotting (p53, cleaved PARP, cytochrome c, and Bax), and ELISA (caspase 9) analysis. Overall, C1 was more cytotoxic, increased the ROS production in dichlorodihydrofluorescein diacetate assay, and induced apoptosis in breast cancer cells. These results illustrate that berry bioactive compounds have strong chemopreventive potential. In this article, we provide information on prooxidant and anticancer activities of *Rubus* bioactive compounds. Natural products have always demonstrated a significant contribution to the development of several cancer chemotherapeutic drugs. Most of these compounds are known to affect the redox state of the cell; and studies on these compounds have focused on their antioxidant property instead of prooxidant properties.

## 1. Introduction

Cancer is the leading cause of death in both developing and developed countries. Globally, cancers of the lung, breast, colon/rectum, and prostate are the most common types. Breast cancer is the most predominant, hormone-associated malignancy in women. The prevalence of breast cancer is growing in developing countries. Upregulation of growth hormone receptors such as estrogen in breast cells is the key reason and the stimulating factor for the development of breast cancer [1].

Historically, plants have been used for many health benefits. About 80-85% of worldwide population rely on traditional plant-based medicines for their health care needs. A number of plant extracts, isolated compounds, and their analogues have been used as effective anticancer drugs, and there has been an increasing interest in the study of therapeutic properties of plant-derived compounds [2]. The characterization and analysis of therapeutic values of plant extracts and the isolated bioactive compounds are a growing area of research. Epidemiological studies show that diets rich in plant-based foods protect against many diseases including cancer. Among the bioactive compounds of plants, phenolics and flavonoid compounds are known to have cytotoxic properties against various tumor cells with low toxicity towards normal cells. Oxidative stress is a normal phenomenon. Normally, the intracellular levels of reactive oxygen species (ROS) are maintained low. Therefore, oxidative stress can be observed as an imbalance between prooxidants and antioxidants [3]. Some of the antioxidants act as prooxidants by inducing nuclear damage and lipid peroxidation if transition metal is available. The number of free OH substitutions initiates the prooxidant activity of a flavonoid. The OH exchange is essential for antioxidant properties, but the more OH substitutions, the stronger prooxidant activities [4].

Raspberries are excellent sources of vitamins such as ascorbic acid. They have been used in traditional and alternative medicine for various illnesses. Some antioxidants like ascorbic acid have both prooxidant and antioxidant effects depending upon the dose. Raspberry extracts, individual polyphenols or in conjunction with other compounds, are able to inhibit the proliferation of cancer cells. They have shown antiproliferative effects on human colon, prostate, breast, and oral cancers [5]. The prooxidant/antioxidant activity of carotenes and lycopene has also been found to depend on their interaction with biological membranes and other coantioxidant molecules. At higher oxygen tension, carotenoids tend to lose their effectiveness as antioxidants, whereas the prooxidant action of tocopherol is evident at low oxygen tension [6].

Apoptosis is the most common cell death mechanism that plays a vital role in normal metabolic function. Tumor cells are characterized by uncontrolled multiplication rates and loss of apoptosis. The activation of apoptotic pathways is one of the cell death pathways by which chemotherapeutic agents kill cancer cells. Agents that block or destroy tumor cell proliferation by inducing apoptosis are considered as promising antitumor mediators [7]. Apoptosis is also a gene-regulated cell death mechanism with well-described biological changes [8]. Initiation of apoptosis is the mode of action of numerous compounds employed in cancer chemotherapy. Hence, the compounds that induce apoptosis are likely to be perfect anticancer drugs. Many bioactive compounds from plants inhibit cancer cell growth through induction of apoptosis; therefore, elucidating the mechanism of apoptosis has a significant implication in cancer chemoprevention. In the present study, we examined the apoptosis induction of two bioactive compounds isolated from a *Rubus* species in human breast carcinoma cells. Most phytochemicals induce apoptosis via the initiation of the intrinsic apoptotic pathways, which includes a range of intracellular stimuli. The major players of this pathway include pro- and antiapoptotic proteins. A difference in the ratio of these proteins leads to mitochondrial membrane damage and results in cytochrome c release, which mediates the activation of caspase 9 [9]. Therefore, alleviating or preventing oxidative damage by ROS should be a potential therapeutic target. Many plant-derived natural compounds induce the controlled production of ROS, which initiates cell death in various cancer conditions through apoptotic pathways.

Previous studies have showed that increased Bax expression may induce apoptosis and increased Bcl-2 expression may inhibit apoptosis [10]. Caspases are a major class of proteases which play a crucial role in cell death. Cytochrome c release by the mitochondria is one of the key features of apoptosis. Bcl-2 proteins prevent apoptosis by decreasing the cytochrome c release to inhibit caspase 3 activation. Bax protein, a vital part of the mitochondrial membrane, helps in the transfer of cytochrome c across the membranes thereby forming apoptotic bodies and activates caspase 9 and caspase 3 that eventually leads to apoptosis [11]. Poly (ADP-ribose) polymerase (PARP) is a family of related enzymes, which induces posttranslational modification proteins, that exist in the nucleus and cytoplasm and is involved in DNA damage repair, gene transcription regulation, telomerase activity regulation, and protein degradation. PARP inhibition may be useful in cancer chemotherapy to selectively sensitize cancer cells to DNA damage agents and promote cell death. PARP is cleaved into two fragments, which causes the loss of its function and leads to apoptosis [12, 13].

Berries are rich in bioactive compounds including phenolics, flavonoids, and anthocyanins. Many bioactive compounds of berries possess antioxidant properties and are important in developing drugs. *Rubus fairholmianus*, the Himalayan raspberry, has been examined for its several pharmacological actions both *in vitro* and *in vivo*. The *R. fairholmianus* root extract induced cell death in colorectal, breast, and lung cancer and melanoma cells via caspase-dependent apoptotic pathways [14–16]. However, evidence regarding the anticancer effect of isolated bioactive compounds from *R. fairholmianus* is inadequate.

**Figure 1:**
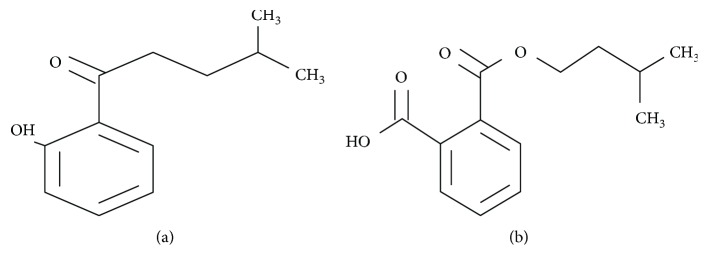
Chemical structure of isolated bioactive compounds from *Rubus fairholmianus*: (a) 1-(2-hydroxyphenyl)-4-methylpentan-1-one (C1) and (b) 2-[(3-methylbutoxy) carbonyl] benzoic acid (C2).

Therefore, the objective of this study was to determine the anticancer effects of the bioactive compounds of *Rubus* such as 1-(2-hydroxyphenyl)-4-methylpentan-1-one (C1) (C_12_H_16_O_2_, 192.25 MW) and 2-[(3-methylbutoxy) carbonyl] benzoic acid (C2) (C_13_H_16_O_4_, 472.53 MW) (Figure 1) on MCF-7 human breast cancer cells *in vitro*.

## 2. Materials and Methods

### 2.1. Extraction and Bioactive Compound Isolation

*Rubus fairholmianus* Gard. wild plants were collected, and a voucher specimen (BSI/SRC/5/23/2010-11/Tech.1657) was deposited in the Botanical Survey of India. Based on the higher radical scavenging activity, root acetone extract was selected for the isolation of bioactive compounds. The roots were extracted successively using polar and nonpolar solvents in a Soxhlet apparatus, and the extract was concentrated to dryness under reduced pressure in a rotary evaporator. *R. fairholmianus* root acetone extract showed higher antioxidant activity, and it was selected for the isolation of bioactive phytochemicals. The preliminary screening was done using thin layer chromatography (TLC) with various solvent combinations; the fractions with similar banding patterns were clubbed and the active fractions obtained were loaded on column chromatography for the compound isolation. The isolated compounds were purified by semipreparative TLC and preparative HPLC. Further ultraviolet, Fourier-transform infrared, nuclear magnetic resonance, and mass spectrometry methods characterized the compounds. Activity-guided column chromatographic isolation yielded 6 compounds [17]; among which, the most active 1-(2-hydroxyphenyl)-4-methylpentan-1-one (C1) and 2-[(3-methylbutoxy) carbonyl] benzoic acid (C2) were selected for the study of induction of cell death in MCF-7 breast cancer cells *in vitro*.

### 2.2. Cell Culture

Human breast cancer cells MCF-7 (ATCC HTB-22) were used for the *in vitro* studies. The cells were grown in Dulbecco's modified Eagle's medium (DMEM) with 10% fetal bovine serum (FBS) (FBS; Gibco 306.00301), supplemented with 1% penicillin/streptomycin (PAA Laboratories GmbH, P11-010) and 1 *μ*g/mL amphotericin B (PAA Laboratories GmbH, P11-001). Human skin fibroblast monolayer cultures (WS1-ATCC CRL1502) were grown in Eagle's minimal essential medium (Invitrogen 32360–026) supplemented with 2 mM L-glutamine (Gibco, 25030), 1 mM sodium pyruvate (Gibco, 11360), 0.1 mM nonessential amino acids (Gibco, 11140), 1% amphotericin B (Gibco, 104813), 1% penicillin-streptomycin (Gibco, 15140), and 10% *v*/*v* fetal bovine serum (FBS; Gibco, 306.00301). Cells were maintained in a CO_2_ incubator (37°C, 5% CO_2_, and 80% humidity) and used as control normal cells. Cells were washed with Hank's Balanced Salt Solution (HBSS, Invitrogen, 10-543F) when it became 80% confluent and detached with TrypLE Express (Gibco, 12604) and subcultured. Cells were seeded at a concentration of 5 × 10^5^ cells/plate (MCF-7 cells) and 6.5 × 10^5^ cells/plate (WS1 cells) in 3.5 cm^2^-diameter culture plates for experimental purposes.

### 2.3. Morphological Analysis

After 24 h of incubation with different concentrations of C1 and C2 (2.5, 5, and 10 *μ*g/mL), the cells were rinsed once with HBSS and replenished with fresh medium, then the morphological changes were carefully noticed using Wirsam Olympus CKX 41 inverted light microscope.

### 2.4. Assessment of Cell Viability, Proliferation, and Cytotoxicity

The effect of C1 and C2 on cell viability was measured by trypan blue assay (Sigma-Aldrich T8154). Ten microliters of cell suspension and 0.4% trypan blue were mixed and added to a haemocytometer and counted using automated cell counter (Countess™ Automated Cell Counter, Invitrogen). CellTiter-Glo^1^ luminescent assay (Promega, G7571, Anatech Analytical Technology, South Africa) quantifies the ATP levels in metabolically active cells. The Cyto-Tox96 X assay (Anatech, Promega G 400) was used to evaluate the cytotoxic activity of C1 and C2 on MCF-7 cells. The membrane integrity and cytotoxicity were assessed by quantifying the lactate dehydrogenase (LDH) released to the culture media following the pretreatment with C1 and C2 bioactive compounds [18].

### 2.5. Annexin V/PI Staining

The Annexin V-fluorescein isothiocyanate (FITC) apoptosis detection kit (Becton Dickinson, 556570, Scientific Group, South Africa) was used to distinguish the population of apoptotic and nonapoptotic cells according to manufacturer's protocol. After the staining process, flow cytometry was performed using Fluorescence-Activated Cell Sorting (FACS) Aria flow cytometer (BD Biosciences) to quantify the population of various cells [18].

### 2.6. Cytochrome c Release and Mitochondrial Membrane Potential Analysis (*ΔΨm*)

An Enzyme-Linked Immunosorbent Assay (ELISA) (Human Cytochrome c Platinum ELISA Kit, Affymetrix eBioscience BMS263) was used to detect cytosolic cytochrome c levels [18]. The evaluation of changes in mitochondrial membrane potential is an indirect measure of intrinsic apoptosis pathway. The *ΔΨm* was analysed using BD™ Mito Screen flow cytometry mitochondrial membrane potential detection kit [18].

### 2.7. DCFH-DA ROS Assay and Hoechst Nuclear Staining

The cellular ROS produced was measured by dichlorodihydrofluorescein diacetate (DCFH-DA) (D-6883, Sigma-Aldrich) assay, and nuclear damage induced by C1 and C2 was observed by Hoechst staining [18].

### 2.8. Immunofluorescence

Cells were cultured in 3.4 cm^2^-diameter culture dishes over sterile cover slips until 80% confluence and treated with C1 and C2 (IC_50_ of C1: 4.69; C2: 8.36 *μ*g/mL) for 24 h. After the treatment, the cells were fixed in 4% formaldehyde for 15 min at room temperature and washed twice with 1x PBS, followed by incubating the cells with permeabilization buffer (0.01% *v*/*v* Triton X-100) for 15 min and then washed twice with 1x PBS. Cells were blocked in PBS containing 3% bovine serum albumin for 1 h, and primary antibody recognizing p53 (Santa Cruz Biotechnology, SC393031), Bax (Santa Cruz Biotechnology, SC493), and caspase 9 (Santa Cruz Biotechnology, SC56076) were added at 1 : 100 dilution for 2 h at room temperature. The cells were washed twice with 1x PBS and FITC (Santa Cruz Biotechnology, SC2010; SC2359) conjugated secondary antibodies were added and incubated for 2 h. The nucleus was stained by DAPI and the slides were examined using the Carl Zeiss Axio Observer Z1 with the filter set 358Ex/461Em.

### 2.9. Caspase 9 Expression by Enzyme-Linked Immunosorbent Assay (ELISA)

We used cell-based ELISA to analyse the expression of caspase 9 in cultured cells. Plates were incubated with 100 *μ*L of 1 : 30 diluted primary antibody (caspase 9, Cat# SC-73548, Santa Cruz Biotechnology), and 100 *μ*L of a 1 : 5000 secondary antibody was added (Goat anti-mouse IgG-HRP: Cat# SC-2005, Santa Cruz Biotechnology), and the colorimetric reaction was measured at 450 nm (Perkin-Elmer, Victor^3^ plate reader) according to the manufacturer's protocol [18].

### 2.10. p53, PARP, Cytochrome c, and Bax Expression by Western Blot Analysis

The protein concentration in cell lysate was assessed using BCA Protein Assay Reagent (Thermo Fisher Scientific, Rockford, IL, USA). Authors used the respective primary antibodies (p53: mouse monoclonal antibody, Cat# SC-99, Santa Cruz Biotechnology; PARP: mouse monoclonal antibody, Cat# SCBSC-8007; Cytochrome c: mouse monoclonal antibody, Cat# SCBSC-13156; Bax: mouse monoclonal antibody, Cat# 336400, Life Technologies; and GAPDH: mouse monoclonal antibody, Cat# MA5-15738, Invitrogen) and the horseradish peroxidase-conjugated secondary antibody (Goat anti-mouse HRP, Cat# SC-2005, Santa Cruz Biotechnology) for the western blot experiments and followed the protocol explained by George et al. [18].

### 2.11. Statistical Analysis

Data were represented as mean ± standard error of the mean (SEM) of at least three tests done in duplicates. The statistical significance was analysed using SigmaPlot version 13.0. The treated groups were compared with the untreated groups by one-way ANOVA to find out the statistical significance. The *p* value less than 0.05 was considered as significant.

## 3. Results

### 3.1. Morphology

**Figure 2:**
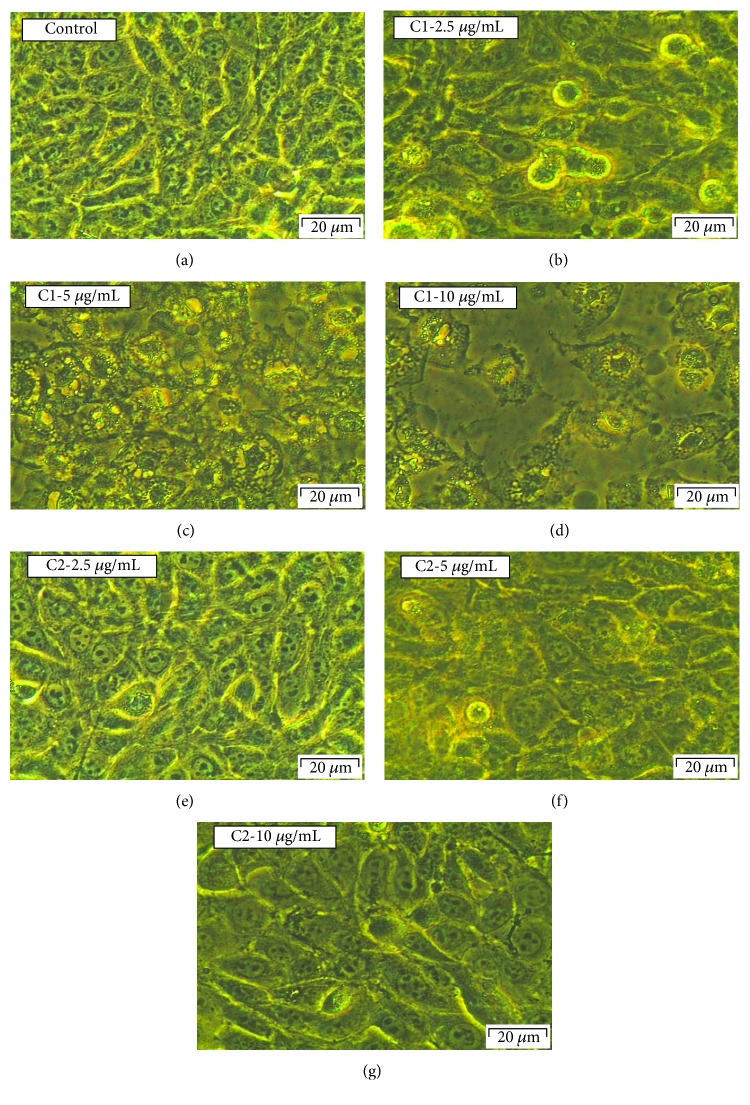
Morphological evaluation of C1- and C2-treated MCF-7 cells. There were no significant visible differences in control (a) and 2.5 *μ*g/mL C2-treated groups (e); the cells did not show any cellular shrinkage and apoptotic bodies after the treatment. However, more dead cells were observed at all concentrations of C1 and higher concentration of C2 (5 and 10 *μ*g/mL). The treated cells showed loss of intact membrane and loss of contact with neighbouring cells and were condensed; being detached from the culture plate showed the features of apoptotic cells.

**Figure 3:**
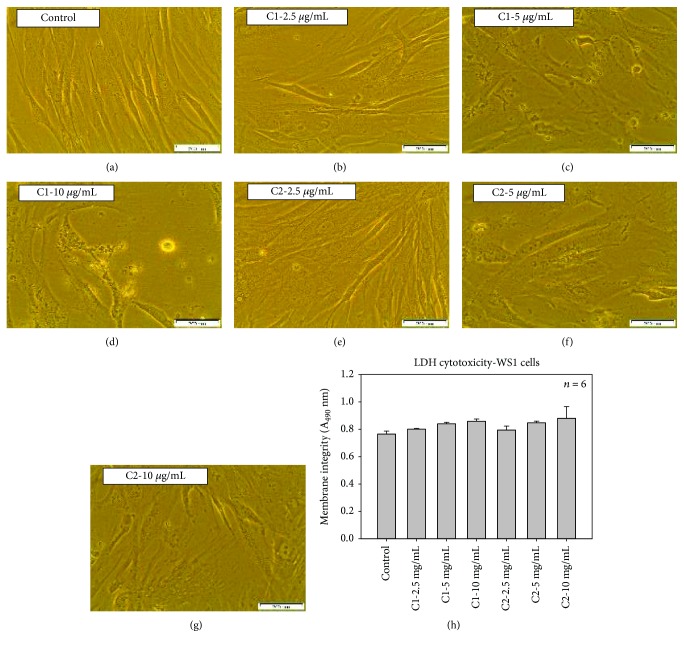
Morphological evaluation of C1- and C2-treated WS1 cells. There were no significant visible differences in control (a) and treated groups (b–g); the cells did not show any cellular shrinkage and apoptotic bodies after the treatment. The lactate dehydrogenase (LDH) cytotoxicity. The LDH cytotoxicity test showed that there is no significant increase in cytotoxicity in WS1 cells after 24 h treatment with C1 and C2 compared to control cells (h).

Morphological variations in MCF-7 cells followed by the treatment with C1 and C2 were compared with control cells (Figures 2(a)–2(g)) and WS1 fibroblast normal cells (Figures 3(a)–3(g)). The pretreated cells presented with an irregular shape, and the number of dead cells in the treated groups was found to be more compared to that in the control group. Total loss of membrane integrity and detachment from the culture plate were observed in the cells treated with C1 and C2. Compared to C2, the C1-treated cells indicated an increased impact with a greater number of dead cells. The most identifiable morphological features of apoptosis such as loss of membrane integrity and cellular shape were observed in the MCF-7 cells treated with bioactive compound C1. The WS1 cells did not show any significant change in morphology after 24 h treatment with C1 and C2.

### 3.2. Viability, Proliferation, and Cytotoxicity

**Figure 4:**
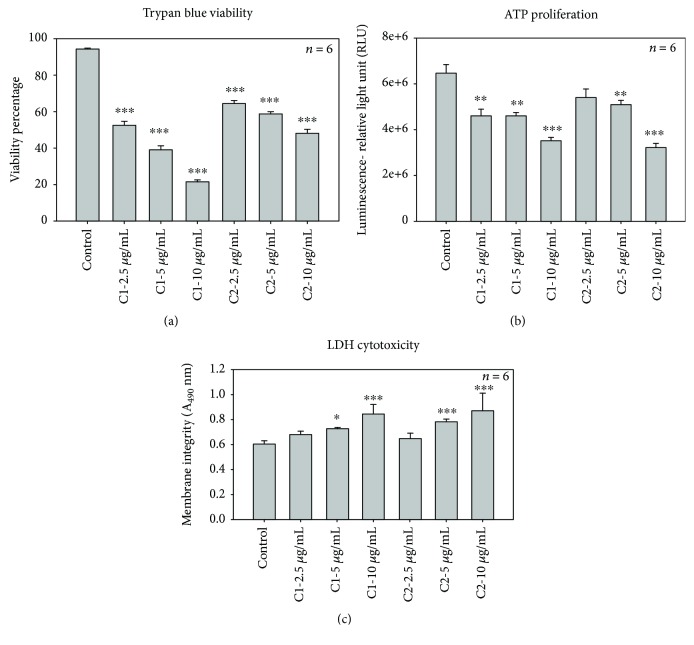
Effect of C1 and C2 on cellular viability, proliferation, and cytotoxicity. (a) Trypan blue viability. Trypan blue viability test showed a significant (∗∗∗*p* < 0.001) decrease in the viability of the MCF-7 cells after C1 and C2 treatment compared to the control cells. (b) The ATP proliferation assay. ATP luminescent cell assay was used to determine MCF-7 cell proliferation after the treatment with C1 and C2. Control cells showed an increased ATP level, whereas a dose-dependent significant (∗∗∗*p* < 0.001 and ∗∗*p* < 0.01) decrease in ATP level was observed in both experimental groups. (c) The lactate dehydrogenase (LDH) cytotoxicity. The LDH cytotoxicity test showed a significant increase in cytotoxicity of cells after the 24 h treatment with C1 and C2 compared to control cells. The significant differences between the treated and control groups are shown as ∗∗∗*p* < 0.001 and ∗*p* < 0.05.

The treatment with C1 and C2 compounds decreased cell viability in MCF-7 cells. We examined the effect of C1 and C2 on cell viability by trypan blue viability test. MCF-7 cells were treated with several concentrations of C1 and C2, and the viability was measured based on the uptake of blue dye and expressed as percentage (Figure 4(a)). Compounds induced a dose-dependent decrease in viability with an inhibitory concentration (IC_50_) of 4.69 and 8.36 *μ*g/mL for C1 and C2, respectively. A significant (*p* < 0.001) reduction in cell number was observed in cells treated with higher concentration of C1 (21.75 ± 0.85%) compared with higher dose of C2 (48.25 ± 2.32%). The decrease in percentage viability was significant with all tested concentrations (*p* < 0.001).

Uncontrolled cell division is a primary contributor to the progression of cancer. The energy levels in MCF-7 cells were high, which was notable from the increased ATP level in untreated cells. Exposure of MCF-7 cells to C1 and C2 leads to the decrease in the intracellular ATP, which shows decreased proliferation rates. We found that the proliferation of MCF-7 cells was inhibited in a concentration-dependent manner after exposure to C1 and C2 (2.5, 5, and 10 *μ*g/mL) (Figure 4(b)). C1 showed relatively high antiproliferative activities on MCF-7 cells compared to C2. However, both compounds were significant in reducing the cellular proliferation compared to untreated cells.

Similar effects were also found when the LDH assay was performed to check the cytotoxic effects of C1 and C2 on MCF-7 cells. The LDH assay was used to measure the cellular membrane integrity following treatment with C1 and C2. The cell membrane damage was measured by LDH release into the culture medium. The WS1 cells (Figure 3(h)) and untreated control cells (Figure 4(c)) displayed reduced LDH release compared to the treated MCF-7 cells. A significant (*p* < 0.001) increase in toxicity was noticed at higher concentrations of C1 and C2 (Figure 4(c)). A 3.08-fold increase in cytotoxicity was observed in MCF-7 cells when treated with C1 at a higher concentration compared to 10 *μ*g/mL of C2 (2.87). Thus, our results suggest that the bioactive compounds isolated from *Rubus* were able to decrease the viability and proliferation in breast cancer cells, while increasing the cytotoxicity of cells after 24 h treatment.

### 3.3. Annexin V/PI Staining

**Figure 5:**
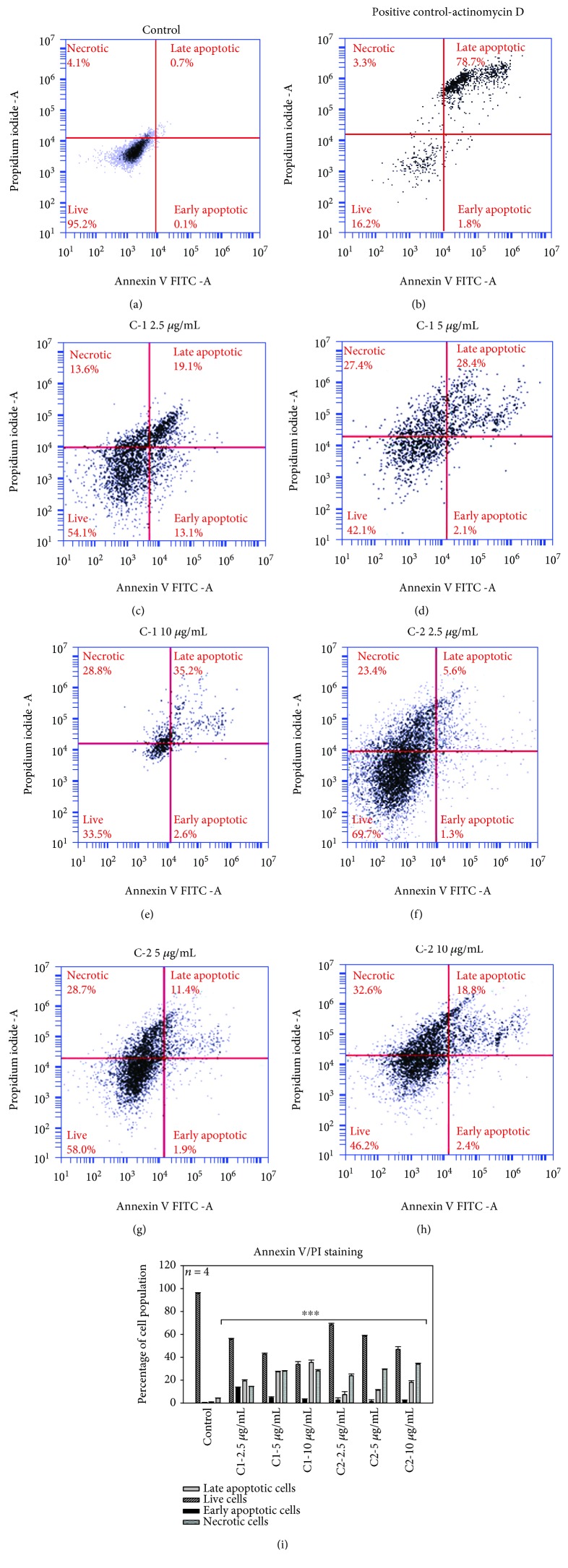
Annexin V FITC/PI staining. Annexin V-PI staining was used to assess the mode of cell death. C1- and C2-treated MCF-7 cells showed an increased percentage of apoptotic cell population after 24 h incubation. The population of early and late apoptotic cell percentage in the control group was found to be lower and the live cell percentage was higher in control cells compared with the experimental groups. A significant decrease (∗∗∗*p* < 0.001) in the live cell and increase in the early and late apoptosis and dead cell percentage were observed in C1- and C2-treated MCF-7 cells. (a–h) Dot plots and (i) the graph with statistical significance.

Apoptosis plays a vital role in the homeostasis, and several morphological and biochemical changes in cells characterize this process. The bioactive compounds C1 and C2 significantly (*p* < 0.001) induced cell death in a dose-dependent manner after 24 h treatment; the Annexin V/PI staining determined the population of apoptotic and nonapoptotic cells. The results of staining displayed a substantial increased uptake of Annexin V by an increased cell population percentage in the lower and upper right quadrants in the pretreated groups (Figures 5(a)–5(h)). C1 and C2 induced early cell apoptosis in MCF-7 cells (2.95 ± 68 and 1.97 ± 0.76%) at the highest concentrations. Flow cytometric analysis of cells displayed that there is a substantial increase in the percentage of late apoptotic cells treated with C1 and C2 (35.45 ± 2.13% and 18.08 ± 1.4%) at 10 *μ*g/mL concentrations, indicative of apoptotic cell death (Figure 5(i)). In contrast, the number of early and late apoptotic and necrotic cell concentrations in untreated cells was found to be very low compared to C1 and C2 treatments.

### 3.4. Cytochrome c Release

**Figure 6:**
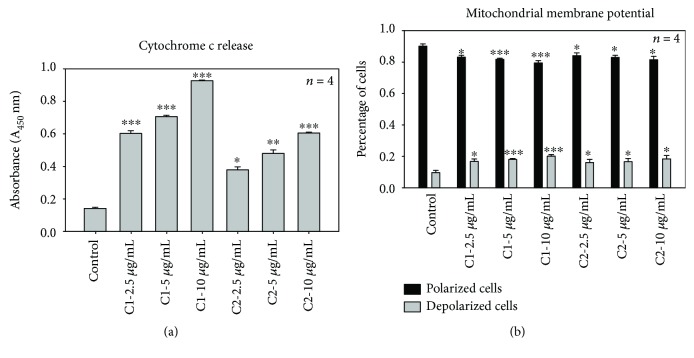
Effect of C1 and C2 on cytochrome c release and mitochondrial membrane potential. (a) Cytochrome c release. Cytochrome c release is an important measure of cellular damage. C1-treated cells showed significant (∗∗∗*p* < 0.001) release of cytochrome c compared to C2 and control cells. The significant differences between the treated and control groups are shown as ∗∗∗*p* < 0.001, ∗∗*p* < 0.01, and ∗*p* < 0.05. (b) Mitochondrial membrane potential. Mitochondrial membrane potential evaluation using the flow cytometric analysis of JC-1 fluorometric stain. Percentages of polarized (black) and depolarized (grey) mitochondrial membrane potential were determined and compared to the percentage of the corresponding control cells. C1- and C2-treated cells showed a significant change in mitochondrial membrane potential (∗∗∗*p* < 0.001).

Cytochrome c release from mitochondria is a critical event in cell death via the intrinsic apoptotic pathway. A difference in various apoptotic and nonapoptotic proteins in cells leads to damage of the mitochondrial membrane resulting in discharge of cytochrome c and followed by caspase activation. The cytochrome c release was determined after 24 h treatment with C1 and C2. The ELISA results of cytochrome c release revealed that in the control, the cells were unable to initiate such a damaging event and low cytochrome c release was observed (Figure 6(a)). All doses of C1-treated cells showed significant (*p* < 0.001) release of cytochrome c compared to C2. However, the treatment with C1 and C2 initiated cell damage in MCF-7 cells and led to an increased cytochrome c release.

### 3.5. Mitochondrial Membrane Potential

To further confirm that C1 and C2 induced apoptosis, mitochondrial membrane potential or mitochondrial destabilization was measured in MCF-7 cells after treatment with C1 and C2.  Figure 6(b) shows that both compounds induced significant damage to mitochondria after 24 h incubation. Compared to C2, C1 showed highly significant results. The percentages of polarized and depolarized membrane potential in each group were determined and compared to the respective percentage of the control cells. After 24 h of incubation with JC-1 stain, no change in membrane potential was detected in control cells. However, changes in both polarized (black) and depolarized (grey) cell populations were noticed in C1- and C2-treated cells. The treated cells increased the depolarized mitochondrial membrane and decreased the polarized membranes. The higher concentrations of C1 (5 and 10 *μ*g/mL) significantly (*p* < 0.001) increased the depolarized mitochondrial membrane and decreased the polarized mitochondrial membrane compared to C2 (*p* < 0.05).

### 3.6. ROS Production and Nuclear Damage

**Figure 7:**
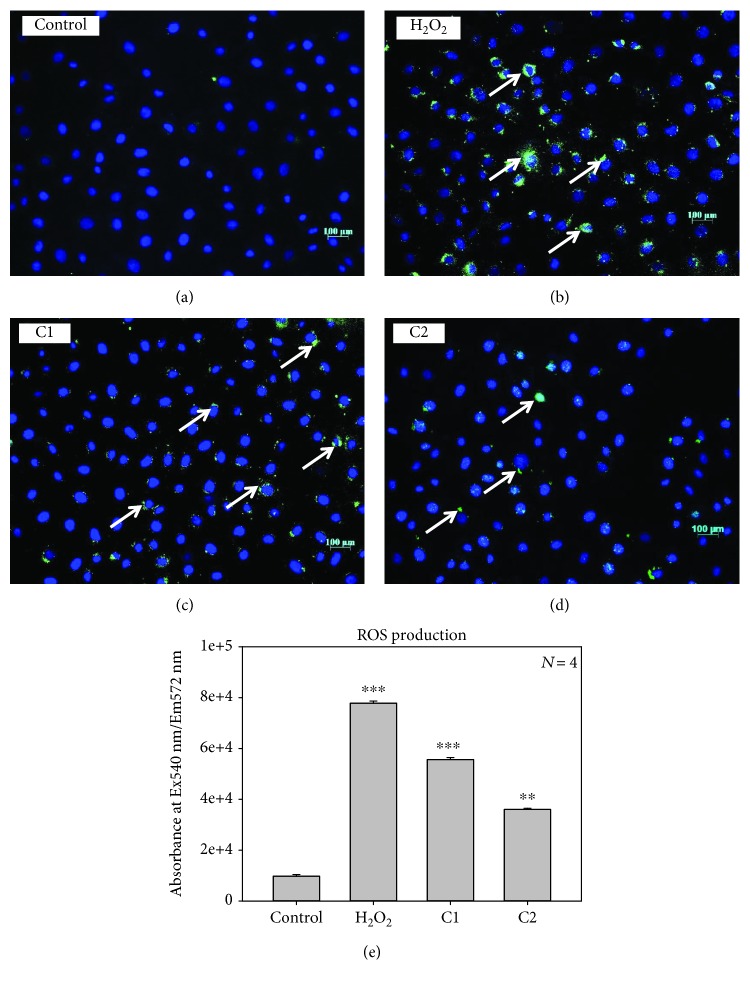
Effect of C1 and C2 on ROS production. ROS production were analysed by DCFH-DA staining in MCF-7 cells: (a) control; (b) positive control: H_2_O_2_; (c) C1: 4.69 *μ*g/mL treated; (d) C2: 8.36 *μ*g/mL treated. The green color indicates the fluorescence of detected ROS production and blue color indicates the nuclear stain-DAPI. C1-treated cells showed increased ROS production than C2-treated cells. (e) Quantitative ROS production analysis using ELISA showing significant ROS production upon treatment with C1 (∗∗∗*p* < 0.001) and C2 (∗∗*p* < 0.01).

The ROS generation was evaluated after 20 h exposure to C1 and C2. A significant increase in the level of intracellular ROS was observed in fluorescent and ELISA methods, as shown in Figures 7(a)–7(e). However, the H_2_O_2_-treated group showed the highest ROS production compared to other groups. This finding indicates that the ROS production is enhanced by the treatment with C1 and C2, which could be one of the reasons behind the cell death. In the quantitative ROS production analysis, using ELISA also showed the increased production when the cells were treated with C1 bioactive compound compared to C2, H_2_O_2_ used as the positive control in these experiments. Some of the important features of apoptosis such as nuclear condensation and DNA damage were examined by Hoechst nuclear staining.

**Figure 8:**
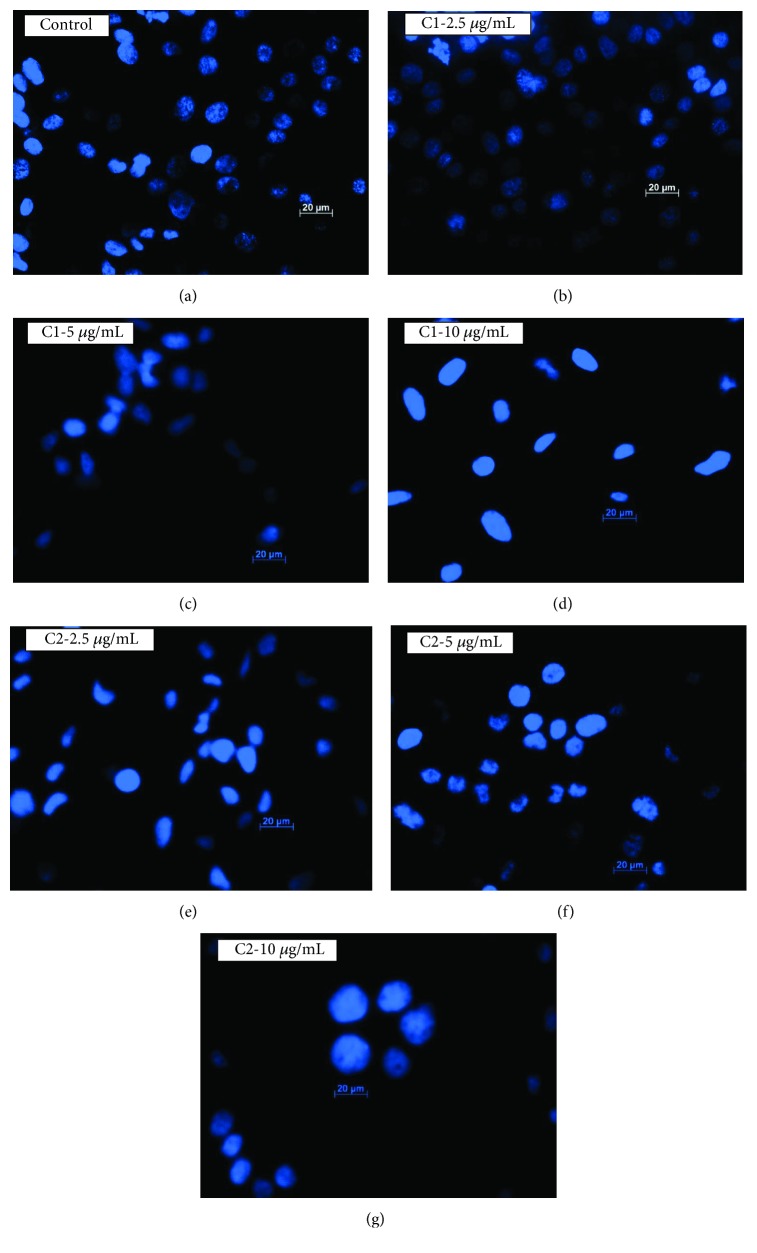
Hoechst staining. Nuclear damage was analysed by Hoechst stain. Control cells (a) showed intact nucleus without any damage; however, the cells treated with C1 and C2 (b–g) at different concentrations showed highly condensed chromatin granules, loss of nuclear shape which indicates the DNA damage. C1-treated cells showed higher nuclear damage.

The Hoechst nuclear stain was used to measure the DNA damage after the treatment with C1 and C2. Results from the control group showed dense spherical and homogenously blue-stained nuclei, while cells treated with C1 and C2 (2.5, 5, and 10 *μ*g/mL) showed irregular-shaped nucleus, suggestive of nuclear condensation as shown in Figures 8(a)–8(g). The treated cells showed shrinkage of chromatin granules with smaller nucleus and scattered nuclear granules, with irregular shape, indicative of nuclear fragmentation. Hence, it suggests that the treatment with C1 and C2 induces apoptotic cell death in MCF-7 cells, as the Hoechst stain was able to show the nuclei changes, which were characteristics of apoptosis.

### 3.7. C1 and C2 Treatment Upregulates Apoptotic Proteins

**Figure 9:**
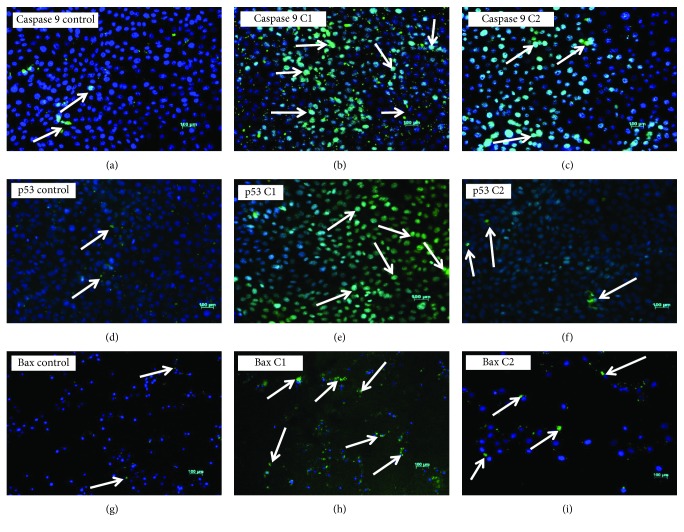
Immunofluorescence: caspase 9, p53, and Bax. (a) Control cells showing very few caspase 9-positive cells; (b) C1 4.69 *μ*g/mL treated showing higher caspase 9-positive cells; (c) C2 8.36 *μ*g/mL treated showing medium caspase 9-positive cells. (d) Control showing very few nuclear staining for active p53; (e) C1 4.69 *μ*g/mL treated showing strong nuclear staining for active p53; (f) C2 8.36 *μ*g/mL treated showing moderate nuclear staining for active p53. (g) Control showing weak staining for active Bax; (h) C1 4.69 *μ*g/mL treated showing strong cytoplasmic staining for active Bax; (i) C2 8.36 *μ*g/mL treated showing moderate cytoplasmic staining for active Bax. C1-treated cells showed increased expression of caspase 9, p53, and Bax proteins than C2-treated cells (×20, original magnification; arrow indicates the presence of proteins; nuclear stain: DAPI (blue color); p53 and Bax protein stain: FITC (green color)).

To investigate the effect of C1 and C2 in inducing the intrinsic apoptotic pathway, we examined the expression of caspase 9, p53, and Bax proteins (Figures 9(a)–9(i)) by immunofluorescence. The apoptotic protein levels will be low in cancer cells compared with the normal dying cells. In our experiments, the apoptotic proteins such as caspase 9, p53, and Bax expressions were high in treated groups than control cells. Upon treatment, active p53 and caspase 9 were highly expressed in the nucleus whereas the activated Bax was highly expressed in the cytoplasm. Bax activation leads to mitochondrial permeability; this was supported by the mitochondrial membrane potential assay and cytochrome c release. Among the bioactive compounds, C1-treated groups showed higher expression of all the four proteins tested than the compound C2.

### 3.8. ELISA and Western Blotting Analysis of Apoptotic Proteins

**Figure 10:**
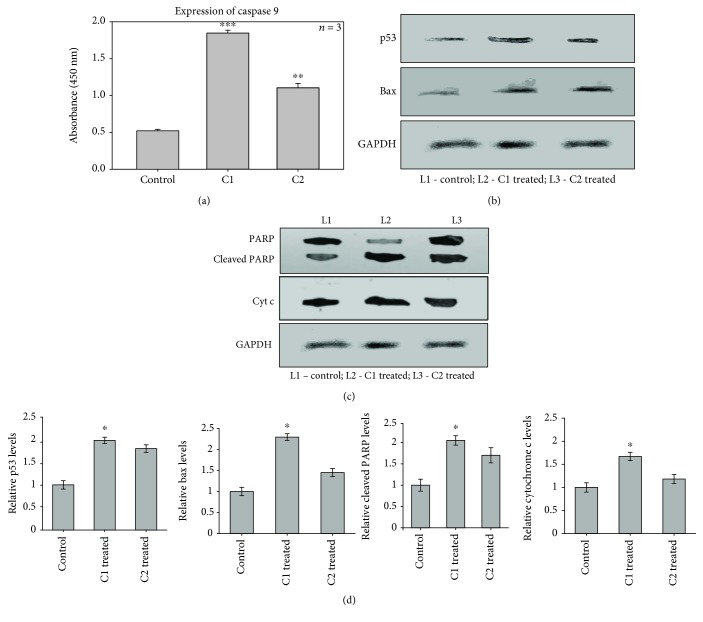
Expression of apoptotic proteins. (a) The effect of C1 and C2 on the expression of caspase 9 by ELISA. (b, c) The effect of C1 and C2 on the expression of p53, Bax, cleaved PARP, and cytochrome c by western blotting (L1: control; L2: C1 treated; L3: C2 treated). (d) The relative expression levels of p53, Bax, cytochrome c, and cleaved PARP. The results showing that apoptotic proteins such as caspase 9, p53, Bax, cytochrome c, and cleaved PARP levels were significantly increased in C1-treated groups than C2 and control cells.

To investigate the effect of C1 and C2 in inducing the intrinsic apoptotic pathway, we examined caspase 9 expression by ELISA (Figure 10(a)) and the expression of p53, Bax, PARP, and cytochrome c by western blotting (Figures 10(b)–10(d)). In cancer cells, the apoptotic protein level will be low compared with apoptotic cells. In our experiments, the expression of apoptotic proteins such as p53, Bax, cleaved PARP, and caspase 9 levels was high in treated groups than control cells, after the treatment with C1 and C2, which favours the apoptosis in MCF-7 cells. The C1-treated groups showed significant (*p* < 0.05 and *p* < 0.001) expression of all four proteins tested. Upon treatment, active p53, Bax, cleaved PARP, cytochrome c, and caspase 9 were highly expressed. The activation of these proteins leads to mitochondrial permeability; these results were supported by the mitochondrial membrane potential, cytochrome c release assay, and immunofluorescence.

## 4. Discussion

Dysregulation of normal apoptotic mechanisms favours cancer cell growth. The dysregulated apoptotic pathways such as downregulation of death receptors and p53 mutations lead to increased incidence of breast cancer. Breast cancer treatments such as chemo, radiation, and hormone therapy induce various apoptotic mechanisms to initiate cell death. Therefore, stimulation of apoptotic mechanisms in breast cancer cells could be an effective way to eradicate breast cancer. This study investigates the antitumor potential of two bioactive compounds C1 and C2 from *R. fairholmianus*. Our previous works described the antiproliferative activities of *Rubus* crude extracts on colorectal, breast, and lung cancer and melanoma cells [14–16]. However, the activities of isolated bioactive compounds from this species [17] against cancer cell lines have not been studied. Previously, many researchers made efforts to understand the effect of plant-derived compounds in induction of cancer cell death [7, 19, 20].

Our data illustrates that the compounds C1 and C2 have antiproliferative and apoptosis inducing effects on MCF-7 human breast adenocarcinoma cells. We pretreated MCF-7 cells with various concentrations of C1 and C2 for 24 h and counted the number of dead and viable cells in order to study the cell death. The cell number of MCF-7 cells was reduced significantly upon treatment with C1 and C2. The results of trypan blue dye exclusion assay demonstrated that both compounds reduced the viability of MCF-7 cells with C1 exhibiting prominent activity. Both compounds induced morphological variations, such as rounding up of cells. In addition, cells treated with compounds revealed loss of membrane integrity and cell detachment from the culture plate. The induced cell death has been categorized as apoptotic or necrotic [21, 22]. The major noticeable feature, which differentiate apoptosis from necrosis, is the presence of apoptotic bodies [23, 24]; these morphological features were observed in MCF-7 cells after treatment, whereas the WS1 fibroblast normal cells did not show any cytotoxicity and significant morphological changes upon treatment with bioactive compounds. This specific cell death induction was further confirmed as the apoptosis-inducing effect of C1 and C2 on MCF-7 cells.

The use of plant-based products is a novel approach in advanced cancer prevention and treatment. The plant-derived compounds require comparatively low cost and cause nontoxic effects with several molecular targets in chemoprevention of cancer [25, 26]. The inhibition of cell proliferation by C1 and C2 was determined using the metabolic ATP proliferative assay. This could either be the result of inhibition of cell growth or a direct cytotoxic effect. The cytoplasmic enzyme LDH will remain within the healthy cells with an intact membrane, but LDH will be released from the membrane-damaged cells. LDH has been established as a biomarker of cell death analysis. In the culture medium, LDH activity increases when cells are treated with cytotoxic agents and they induce apoptosis or necrosis [27–29]. The LDH cytotoxicity assay directly measures the toxicity by evaluating the LDH released upon the treatment with C1 and C2. Apoptosis and related cellular mechanisms display intense effects on cancer cell progression; those events are perfect target in several cancer therapies [30]. The role of natural products has been broadly examined for its involvement in cancerogenesis and metastasis [31].

Evaluation of apoptosis further confirmed that pretreatment with C1 and C2 induced cytochrome c release and drop in mitochondrial membrane potential. The loss in mitochondrial membrane integrity is related with intrinsic apoptosis [32]. When the death signal starts in a cell, the C terminal sequence of the receptor activates that targets the mitochondrial outer membrane and leads to permeabilization, which finally leads to the cell death [33]. The decrease of mitochondrial membrane potential suggests the initiation of the mitochondrial apoptotic pathway. Depolarization of mitochondrial membrane is due to the degeneration of mitochondrial membrane potential and interruption of the electron transport chain (ETC) gradient. The shift in mitochondrial polarization when exposed to the compounds could be the result of intact ETC [34]. The dose-dependent incubation with C1 and C2 depicted a continuous reduction of mitochondrial membrane potential.

The upregulation of Bax proteins may lead to cytochrome c release and thus caspase 9 upregulation [35]. The biochemical analysis of MCF-7 cells after the treatment with C1 and C2 confirmed such hypothesis. Hence, the viability, cytotoxicity, and proliferative results in conjunction with the mitochondrial dysfunction and cytochrome c release demonstrated the initiation of apoptotic pathway by C1 and C2.

ROS are either free radicals or reactive anions with oxygen ions and peroxides [36]. High level of ROS can destroy the integrity of the plasma membrane and cause DNA damage, cumulatively known as oxidative stress [37, 38]. Interestingly, we observed that ROS production in C1- and C2-treated MCF-7 cells was higher compared to that in untreated cells. The accumulation of ROS disturbs the redox control of cell cycle progression via phosphorylation and ubiquitination of cell cycle proteins, leading to aberrant cell proliferation and apoptosis [39]. Although ROS is dangerous to cells, the anticancer role of various treatments depends on their ability to stimulate controlled ROS production, which changes cellular redox balance leading to oxidative stress, damage to mitochondria, and consequent apoptosis induction [40]. Our results also supported this fact by mitochondrial destabilization, by the increased ROS production, which in turn regulated the expression of apoptotic proteins.

Several studies have revealed that mitochondria has a potential role in apoptosis [41, 42]. The mitochondrial membrane potential variations lead to the release of apoptogenic factors including cytochrome c. Cytochrome c forms an apoptosome with procaspase 9, apoptotic protease activating factor-1 (Apaf-1), and ATP. The apoptosome further activates downstream caspase signals [43, 44]. Initiator caspase 9 is activated during cytochrome c release [44], which can further activate executioner caspases and achieve apoptosis [45]. Treatment with C1 and C2 causes a dose-dependent activation of caspase 9; these results propose that C1 and C2 induced apoptosis via the mitochondrial intrinsic pathway. Mitochondrial molecules involved in ROS generation and pro- and antiapoptotic factor regulation have been reported to control apoptosis in several cell lines [46]. Studies show that ROS can facilitate mitochondrial membrane permeabilization [47]. The ROS produced in oxidation processes are essential to living organisms to produce the energy required to fuel biological processes. However, excessive production of ROS damages the cells because ROS destroy molecules such as DNA and proteins. Thus, ROS play an important role in the pathogenesis of various serious diseases, such as neurodegenerative disorders, cancer, cardiovascular diseases, atherosclerosis, cataracts, and inflammation [48–51].

Deregulation of pro- and antiapoptotic factors disrupts mitochondrial role, leading to cytochrome c release to the cytoplasm and subsequent activation of caspase cascade [52]. In our experiments, treatment with C1 and C2 resulted in the expression of Bax and release of cytochrome c in MCF-7 cells. Bax possibly controls the cytochrome c release from mitochondria that occurs after the treatment. ROS also plays a crucial role in the modulation of proapoptotic Bax and antiapoptotic Bcl2, and studies reported that the translocation of Bax to mitochondria could change permeability [53, 54]. Based on our results, it is possible that sufficient Bax exists in the mitochondrial membrane to induce cytochrome c release and intrinsic apoptosis after the treatment with C1 and C2.

p53 is a multifunctional tumor suppressor that regulates DNA repair, cell cycle arrest, apoptosis, and cell survival as well as oxidative stress. p53 responds to a wide range of cell death stimuli, which can induce apoptosis by activating gene expression or by permeabilizing the mitochondria. Increased ROS in cancer cells is also associated with the activation of a key signaling protein p53. p53 accumulates in the nucleus and controls the expression of proapoptotic members, Bax and PUMA. Once upregulation of PUMA occurs, PUMA binds to Bcl-xL, releasing p53 to activate Bax [55]. p53 can translocate to mitochondria, interact with the antiapoptotic Bcl-2 family of proteins, neutralise them, and cause apoptosis [56]. The present study showed the expression of p53 in MCF-7 cells when treated with C1 and C2; therefore, the induction of apoptosis in MCF-7 cells upon *Rubus* bioactive compound treatment is a major target for cancer therapy.

Relatively high PARP expression was found in triple-negative breast cancers (TNBC) [57]. The findings from this research suggest that inhibition of PARP could be a potential therapeutic approach in breast cancer and other tumor types. Cleavage of PARP to its inactive form is considered as an important event in cancer cell apoptosis [57]. PARP cleavage and higher expression of cleaved PARP in the C1- and C2-treated groups and the generation of intracellular ROS assured the induction of apoptosis. Generation of intracellular ROS plays a pivotal role in apoptosis [37]. In this study, upregulation of Bax with higher expression of p53, caspase 9, cytochrome c, and PARP provides an insight that C1 and C2 could initiate a favourable signal transduction mechanism, which triggers MCF-7 cells to undergo apoptosis. It is implicated that caspase 3 and caspase 9 are the major signaling proteins in inducing apoptotic cascade. Cleavage and higher expression of downstream protein PARP also established the DNA-related damage in MCF-7 cells [58].

Several studies have shown that bioactive compounds such as polyphenols and flavonoids induce cell death via apoptosis [59–62] with numerous morphological and biochemical changes. Evidence suggests that berries have beneficial effects against numerous cancers; its anticancer potential has been related to a multitude of bioactive compounds, including polyphenols, stilbenoids, lignans, and triterpenoids. Reports show that the antiproliferative effects of berry compounds are partially mediated through their ability to repair damage from oxidative stress. Bioactive compounds from berries also regulate carcinogen and xenobiotic metabolizing enzymes, various transcription factors, and cellular signaling pathways and apoptosis. They may also potentially sensitize tumor cells to chemotherapeutic agents by inhibiting pathways that lead to treatment resistance [63].

Compounds 1-(2-hydroxyphenyl)-4-methylpentan-1-one (C1) and 2-[(3-methylbutoxy) carbonyl] benzoic acid (C2) were isolated from *R. fairholmianus* root extract by George et al. [17] which can be used as a promising lead compound for the development of potent drug for the prevention and treatment of malignant tumors. The studied compounds markedly suppressed the proliferation of MCF-7 cells and induced cytotoxicity, which favours the induction of apoptosis by cytochrome c release and altering mitochondrial membrane potential significantly. Taken together, the results from this study preliminarily demonstrated that the compounds isolated from *Rubus* species (C1 and C2) induce apoptosis in human breast cancer cells by causing accumulation of intracellular ROS, by activation of mitochondrial signaling pathway by showing higher expression of cleaved PARP, caspase 9, cytochrome c, and Bax apoptotic proteins. We provide insights on and perspectives for future development of effective therapeutic ROS-inducing anticancer agents from plant extracts. In this article, we present evidence on the role of ROS in inducing cancer cell death, which may be utilized to increase our understanding on ROS-associated signaling pathways in cancer chemotherapy. ROS-mediated cell death induction reported in this article will be verified more accurately in our future investigations analysing ROS in the presence of antioxidants.

**Figure 11:**
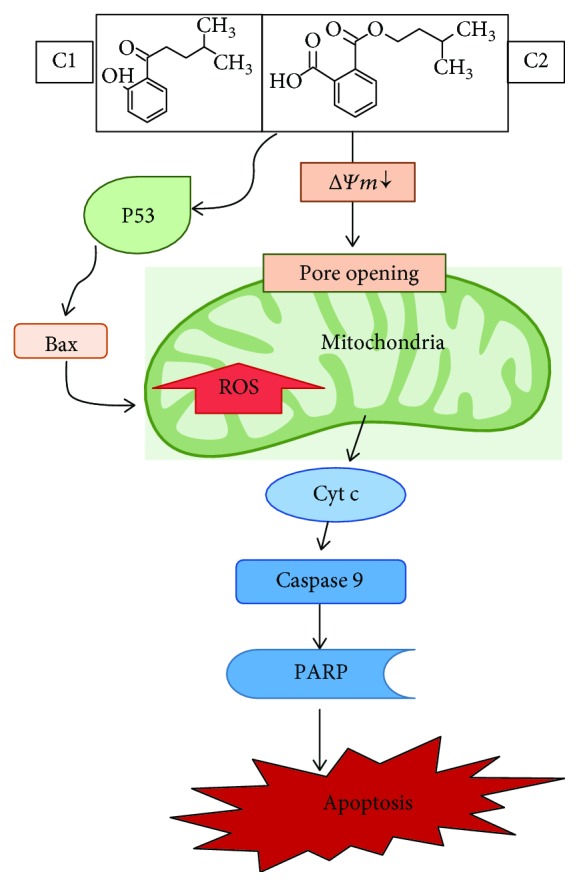
Proposed cell death mechanism. An illustration of proposed cell death induced by C1 and C2 in MCF-7 cells. Mitochondria mediated caspase-dependent intrinsic apoptotic pathway is upregulated. Increased expression of p53, caspase 9, cleaved PARP, and Bax proteins mediated the cell death.

The upregulation of apoptotic proteins (p53, Bax, cytochrome c, PARP, and caspase 9) were significantly observed after the treatment with *Rubus* bioactive compounds. The proposed mechanism by which ROS mediated cell death induction by C1 and C2 bioactive compounds from *Rubus* is illustrated in Figure 11. In conclusion, a novel mechanism of bioactive compounds from *Rubus* involves the induction of apoptotic cell death via caspase activation. The results of this study are consistent with the notion that bioactive compounds of *Rubus* are beneficial in preventing cancer cell proliferation. Hence, this article provides an outline of *Rubus* prooxidant and anticancer properties, with the special focus on C1 and C2, and discusses their possible use as breast cancer chemotherapeutic agents. Further studies are recommended to evaluate the effect of these compounds on other mode of cell death mechanisms.

## Data Availability

The datasets generated during and/or analysed during the current study are available from the corresponding author on reasonable request.

## Supplementary Materials

Supplementary Materials Supplementary Figure 1: LDH cytotoxicity of C1- and C2-treated A549 and A375 cells. Lung cancer cells (A549-ATCC CCL185) were cultured in Roswell Park Memorial Institute 1640 medium complemented with 10% FBS, 0.5% penicillin-streptomycin, and 0.5% amphotericin B. The melanoma cells (A375-ATCC CRL1619) were cultured in Dulbecco's modified Eagle's media with 1.2 g/L sodium carbonate, 10% FBS, 10 mM nonessential amino acids, 0.5 mM sodium pyruvate, 2.5 mM L-glutamine, 1% penicillin-streptomycin, and 1% amphotericin B. Once the cells reached 80% confluence, they were seeded in a 3.5 cm^2^-diameter culture plates at a concentration of 2 × 10^5^ (A549) 5 × 10^5^ (A375) cells for experimental purposes. The cultures were incubated at 37°C with 5% CO_2_ and 85% humidity. The Cyto-Tox96 X assay (Anatech, Promega G 400) was used to evaluate the cytotoxic activity of C1 and C2 on A549 cells. The cytotoxicity assay results showed that both C1 and C2 significantly induced the release of LDH from A549 and A375 cells in a dose-dependent manner indicating its cytotoxicity; however, these bioactive compounds found to be more toxic towards A549 lung cancer cells compared to A375 melanoma cells.
