# Supplementary material for: Increased Oxidative Stress Induced by Rubus Bioactive Compounds Induce Apoptotic Cell Death in Human Breast Cancer Cells
Source: Oxid Med Cell Longev. 2019 Jun 3;2019:6797921. doi: 10.1155/2019/6797921 (PMC6589211; doi:10.1155/2019/6797921)
Supplement: Supplementary Materials — Supplementary Figure 1: LDH cytotoxicity of C1- and C2-treated A549 and A375 cells. Lung cancer cells (A549-ATCC CCL185) were cultured in Roswell Park Memorial Institute 1640 medium complemented with 10% FBS, 0.5% penicillin-streptomycin, and 0.5% amphotericin B. The melanoma cells (A375-ATCC CRL1619) were cultured in Dulbecco's modified Eagle's media with 1.2 g/L sodium carbonate, 10% FBS, 10 mM nonessential amino acids, 0.5 mM sodium pyruvate, 2.5 mM L-glutamine, 1% penicillin-streptomycin, and 1% amphotericin B. Once the cells reached 80% confluence, they were seeded in a 3.5 cm2-diameter culture plates at a concentration of 2 × 10^5^ (A549) 5 × 10^5^ (A375) cells for experimental purposes. The cultures were incubated at 37°C with 5% CO2 and 85% humidity. The Cyto-Tox96 X assay (Anatech, Promega G 400) was used to evaluate the cytotoxic activity of C1 and C2 on A549 cells. The cytotoxicity assay results showed that both C1 and C2 significantly induced the release of LDH from A549 and A375 cells in a dose-dependent manner indicating its cytotoxicity; however, these bioactive compounds found to be more toxic towards A549 lung cancer cells compared to A375 melanoma cells. [file 6797921.f1.pptx]

## Slide 1
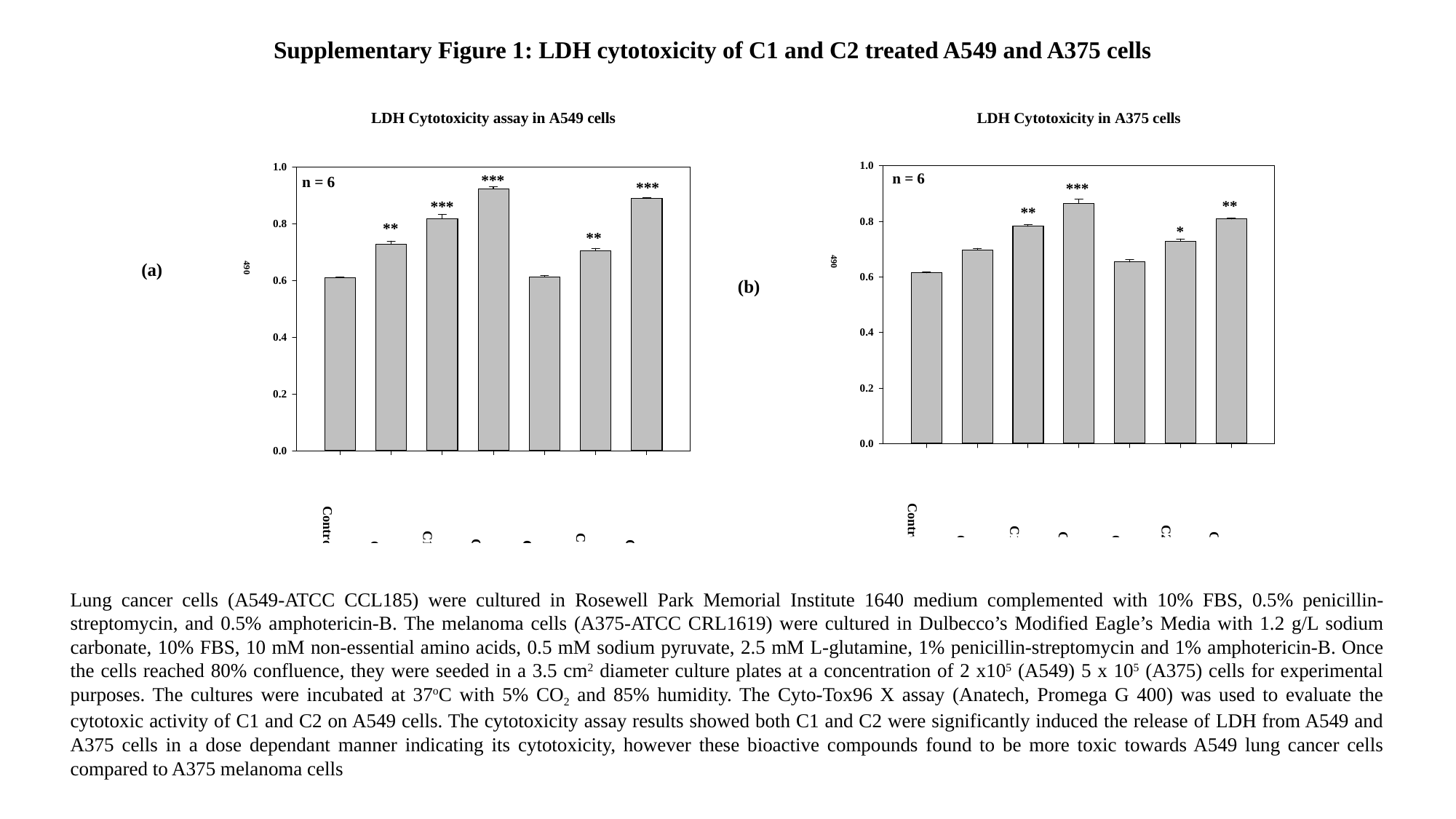

Supplementary Figure 1: LDH cytotoxicity of C1 and C2 treated A549 and A375 cells
(a)
(b)
Lung cancer cells (A549-ATCC CCL185) were cultured in Rosewell Park Memorial Institute 1640 medium complemented with 10% FBS, 0.5% penicillin-streptomycin, and 0.5% amphotericin-B. The melanoma cells (A375-ATCC CRL1619) were cultured in Dulbecco’s Modified Eagle’s Media with 1.2 g/L sodium carbonate, 10% FBS, 10 mM non-essential amino acids, 0.5 mM sodium pyruvate, 2.5 mM L-glutamine, 1% penicillin-streptomycin and 1% amphotericin-B. Once the cells reached 80% confluence, they were seeded in a 3.5 cm2 diameter culture plates at a concentration of 2 x10^5^ (A549) 5 x 10^5^ (A375) cells for experimental purposes. The cultures were incubated at 37oC with 5% CO2 and 85% humidity. The Cyto-Tox96 X assay (Anatech, Promega G 400) was used to evaluate the cytotoxic activity of C1 and C2 on A549 cells. The cytotoxicity assay results showed both C1 and C2 were significantly induced the release of LDH from A549 and A375 cells in a dose dependant manner indicating its cytotoxicity, however these bioactive compounds found to be more toxic towards A549 lung cancer cells compared to A375 melanoma cells
